# Telomere targeting with a novel G-quadruplex-interactive ligand BRACO-19 induces T-loop disassembly and telomerase displacement in human glioblastoma cells

**DOI:** 10.18632/oncotarget.7483

**Published:** 2016-02-18

**Authors:** Guangtong Zhou, Xinrui Liu, Yunqian Li, Songbai Xu, Chengyuan Ma, Xinmin Wu, Ye Cheng, Zhiyun Yu, Gang Zhao, Yong Chen

**Affiliations:** ^1^ Department of Neurosurgery, First Hospital of Jilin University, Changchun, China

**Keywords:** telomere, G-quadruplex, telomerase, DNA damage, T-loop

## Abstract

Interference with telomerase and telomere maintenance is emerging as an attractive target for anticancer therapies. Ligand-induced stabilization of G-quadruplex formation by the telomeric DNA 3′-overhang inhibits telomerase from catalyzing telomeric DNA synthesis and from capping telomeric ends, making these ligands good candidates for chemotherapeutic purposes. BRACO-19 is one of the most effective and specific ligand for telomeric G4. It is shown here that BRACO-19 suppresses proliferation and reduces telomerase activity in human glioblastoma cells, paralleled by the displacement of telomerase from nuclear to cytoplasm. Meanwhile, BRACO-19 triggers extensive DNA damage response at telomere, which may result from uncapping and disassembly of telomeric T-loop structure, characterized by the formation of anaphase bridge and telomere fusion, as well as the release of telomere-binding protein from telomere. The resulting dysfunctional telomere ultimately provokes p53 and p21-mediated cell cycle arrest, apoptosis and senescence. Notably, normal primary astrocytes do not respond to the treatment of BRACO-19, suggesting the agent's good selectivity for cancer cells. These results reinforce the notion that G-quadruplex binding compounds can act as broad inhibitors of telomere-related processes and have potential as selective antineoplastic drugs for various tumors including malignant gliomas.

## INTRODUCTION

Human telomeres which are located on the ends of chromosomes are composed of repetitive TTAGGG sequences and associated proteins. They play important roles in protecting the ends of chromosomes from recombination, end-to-end fusions, or exonuclease action [1–3]. In somatic cells, telomere progressively shortens with every cell division as a consequence of the inability of DNA polymerase to fully replicate the ends [4]. By contrast, telomeres in tumor cells are stably maintained in length and significantly shorter than normal cells. In approximately 85% of human tumors, the integrity of telomere is maintained by a specialized reverse transcriptase named telomerase [5]. In a small percentage of tumors, an alternate pathway for telomere length may be operational and involves recombination events [6]. Therefore, interference with telomerase and telomere maintenance represents an attractive strategy for anticancer therapy [7–9].

As well-known, the problem of traditional strategy for direct telomerase inhibition is the long lag period required before telomeres reach the critically short length required for eventual senescence and apoptosis [4, 7, 10]. Furthermore, it has been reported that in cancer cells, inhibition of telomerase activity might activate a recombination-based alternative lengthening of telomeres (ALT) mechanism for telomere maintenance, which is one of the major limitations for the development of clinically useful telomerase inhibitors [6, 11, 12]. In humans, the telomere is composed of G-rich duplex with a single-stranded (ss) 3′-overhang. The 3′-overhang is either accessible for telomerase extension in an open state or inaccessible in a capped (or closed) conformation that involves the formation of loop motifs, termed as T-loop and D-loop [1, 13]. Uncapping of the telomere ends by different means leads to telomeric dysfunction characterized by end-to-end fusion, inappropriate recombination, anaphase bridges and G-overhang degradation that either result in apoptosis or senescence [1, 14, 15].

It has been reported that when the 3′-overhang of telomeric DNA forms quadruplex structure it cannot be elongated by telomerase [16]. Therefore, small molecules that can induce and stabilize human telomeric G-quadruplex are considered as promising anticancer agents [17, 18], the first example was reported by Sun et al. [19]. This strategy can result in both shortening telomeres and directly causing telomere dysfunction, which would trigger a short-term apoptosis/senescence in human cancer cells [17, 20]. Several types of G4 ligands have been designed to counteract telomerase and telomere for anticancer therapy [17, 18, 20]. One of the most potent G-quadruplex ligands is BRACO-19, a 3,6,9-trisubstituted acridine derivative designed to stabilize the telomeric quadruplex DNA structures [21], which has been shown to inhibit telomerase activity [22] and to display antitumor activity [23]. However, G-quadruplex-interactive molecules acting on telomeres are still in preclinical or early clinical development [17, 18]. Therefore, identification of the underlying mechanisms of action is still a major challenge.

Malignant gliomas are the most common primary tumor of the central nervous systems and represent the second leading cause of cancer-related deaths in children and young adults. The most frequent form, glioblastoma multiforme, is very aggressive and invasive and is highly refractory to anticancer treatment. Glioblastoma patients generally survive for less than 18 months after diagnosis, despite treatment by a combination of surgery, radiation therapy and chemotherapy [24]. Although much less frequent than telomerase activation in most cancer types, it has been suggested that ALT occurs in almost 25% of glioblastoma multiforme tumors [25].

Here we investigated G4 ligand-mediated anti-tumor effects on malignant glioma cells using the acridine derivative BRACO-19 as proof-of-concept and further assessed the effects of BRACO-19 on telomere maintenance and telomerase function. In this article, we demonstrated that BRACO-19 can inhibit telomerase activity, induce telomere uncapping and removal of telomere-binding protein from telomere, resulting in telomere aberration, DNA damage response and cell growth cessation. Moreover, BRACO-19 can cause translocation of telomerase from nuclear to cytoplasm. These findings reveal the telomere-specific effects of BRACO-19 and validate telomeres as promising targets for future anticancer therapies.

## RESULTS

### Exposure to BRACO-19 results in growth inhibition and telomerase activity inhibition in glioma cells

**Figure 1:**
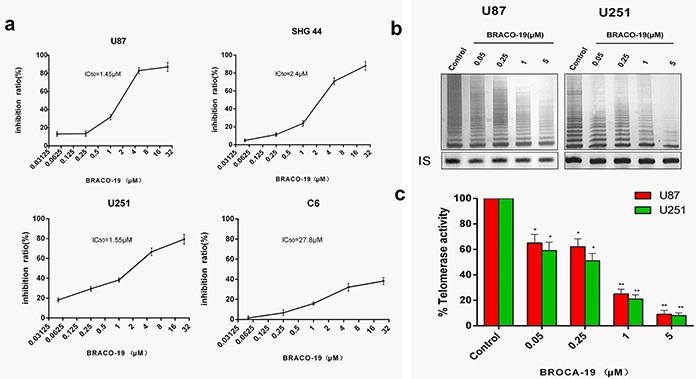
Acute BRACO-19 exposure inhibits proliferation and telomerase activity of high grade brain tumor cells **a.** U87, U251 and SHG44 cells exhibited IC_50_ values of 1.45, 1.55 and 2.5 μM respectively when 0.05–25μM BRACO-19 was used, representing a significant inhibition of cell proliferation (*P*<0.05 for each drug concentration versus untreated). C6 cells exhibited IC_50_ values of 27.8 μM, showed only a modest cytotoxic effect. **b.** Telomerase activity inhibition induced by BRACO-19 in U87 and U251 cells. Cells were treated with increasing concentrations of BRACO-19 for 72 hours, CHAPS extract was prepared and equivalent amounts of protein (500 ng) were subjected to a standard TRAP assay. The position of the internal standard was indicated as IS. **c.** Telomerase activity was quantitated as the percent of the corresponding control sample. The mean of three independent experiments with comparable results was shown. Error bars indicate ± s.d. **P< 0.001, two-tailed student's *t*-test.

Glioma and glioblastoma cells were treated with BRACO-19 for 72 hours at a concentration range of 0.05–25μM. U87, U251 and SHG-44 cells showed a significant dose-dependent cytotoxic effect with IC_50_ values of 1.45, 1.55 and 2.5 μM (Figure 1a), respectively. However, exposure of C6 cells to BRACO-19 showed only a modest cytotoxic effect, with an IC_50_ value of 27.8 μM, approximately 60% of viable cells remained after the highest drug dose treatment for 72 hours (Figure 1a). According to these results, U87, U251 and SHG-44 cells exhibited approximately 20 fold increased sensitivity compared to C6 cells. The following experiments were conducted using U87 and U251 cell lines which possess greater sensitivity. However, BRACO-19 did not induce acute growth inhibition in human normal primary astrocytes (Supplementary Figure S1), suggesting the selective killing for glioma cells over normal cells. The telomerase inhibitory effects of BRACO-19 in U87 and U251 cells were also investigated with the traditional TRAP assay [5, 26–28]. As shown in Figure 1b and 1c, after 72 hours treatment of BRACO-19, a significant dose-dependent telomerase activity inhibition in glioma cells was observed. At 5μM drug concentration, telomerase activity is almost completely inhibited. Meanwhile, no inhibition of telomerase substrate internal control (Taq polymerase activity) was observed at concentrations for telomerase activity inhibition. BRACO-19 also displayed time-dependent telomerase activity suppression in U87 and U251 cells (Supplementary Figure S2). The inhibitory efficiency reached maximum on day 9 after treatment.

### Growth suppression induced by BRACO-19 associated with the production of DNA damage response

**Figure 2:**
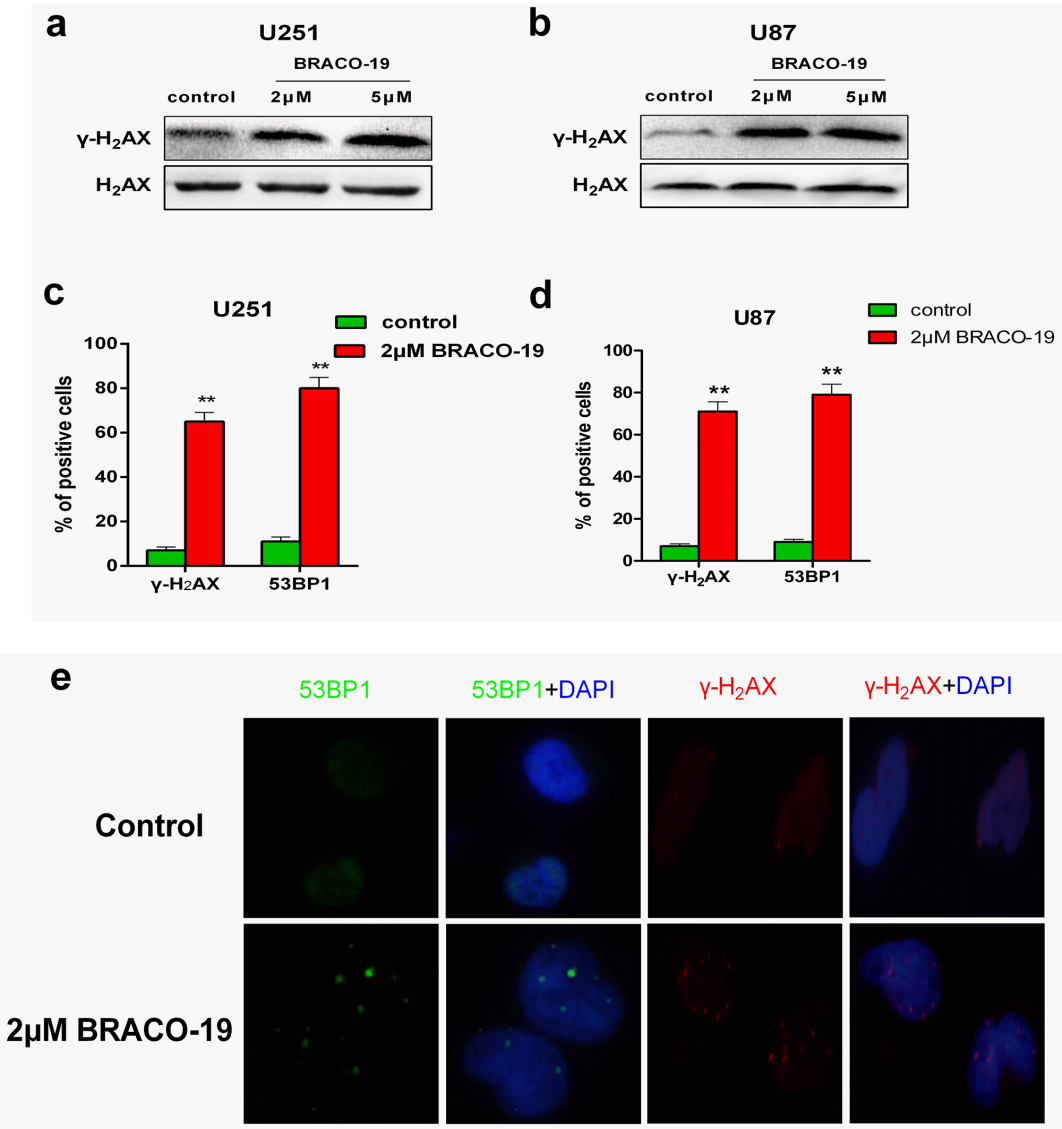
BRACO-19 induces the production of DNA damage response **a, b.** Western blot analysis of γ-H_2_AX in U251 and U87 cells treated with BRACO-19 (2 μM and 5μM) for 72 hours. The levels of H_2_AX were used as loading control. **c, d.** Percentage of cells containing γ-H_2_AX and 53BP1 foci in U251 and U87 cells treated with BRACO-19 (2 μM) for 72 hours. γ-H_2_AX and 53BP1 foci were quantified using mouse monoclonal antibodies. On average, more than 200 cells were screened in three independent experiments. Error bars indicate s.d. **P< 0.001, two-tailed student's *t*-test. **e.** Representative immunofluorescence images of γ-H_2_AX and 53BP1 foci in U87 cells treated with BRACO-19 (2 μM) for 72 hours. Scale bar equals 5 μm.

As reported previously, growth inhibition induced by telomere-targeting agents often associates with the telomere dysfunction and production of DNA-damage response [29, 30]. To investigate whether the treatment with BRACO-19 would induce the production of DNA-damage response, immunoblotting and immunofluorescence experiments were performed in drug-treated U87 and U251 cells. As shown in Figure 2a and 2b, strong phosphorylation of γ-H_2_AX after 72h treatment with BRACO-19 (2μM), which was a hallmark of DNA double-strand break response [27–30], was observed, which was also confirmed by the immunofluorescence for γ-H_2_AX and 53BP1, another DNA-damage response factor [27–30] (Figure 2c–2e). There are more γ-H_2_AX- and 53BP1-positive cells after treatment with BRACO-19 (2μM) compared with untreated groups (Figure 2c–2d, *P* < 0.001). However, γ-H_2_AX foci in cells were not observed in BRACO-19 treated normal primary astrocytes (Supplementary Figure S3), even at longer exposure time (data not shown). Based on these results, we demonstrated that growth inhibition induced by BRACO-19 was tumor cell-specific and associated with the production of DNA damage response.

### DNA-damage response triggered by BRACO-19 occurred at telomere

**Figure 3:**
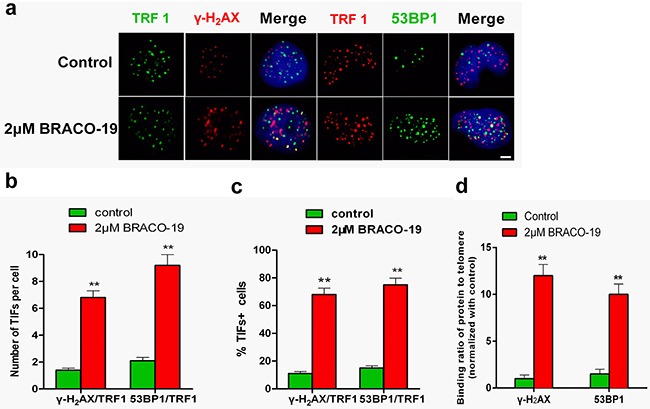
DNA-damage response triggered by BRACO-19 occurred at telomeres **a.** U87 cells treated with BRACO-19 (2 μM) for 72 hours were fixed and processed for immunofluorescence using antibodies against γ-H_2_AX (red)/TRF1 (green) or 53BP1 (green)/TRF1 (red), respectively. Representative confocal images were shown. Scale bar equals 5 μm. **b.** TIF index, defined as foci of DNA-damage response factors that coincided with TRF1, was calculated as the percentage of TIF-positive cells in U87 cells treated with BRACO-19 (2μM). Cells with four or more γ-H_2_AX/TRF1or 53BP1/TRF1 foci were scored as TIF-positive. Error bars indicate s.d. **P < 0.001, two-tailed student's *t*-test. **c.** Average number of TIFs per nucleus in U87 cells treated with BRACO-19 (2μM). Error bars indicated ± s.d. **P < 0.005, two-tailed student's *t*-test. **d.** Binding of γ-H_2_AX and 53BP1 was examined by ChIP assay and detected by qRT–PCR amplification of the telomeric region in U87 cells treated with BRACO-19 (2μM). Data represented triplicate ChIP experiments, each with technical triplicates of qRT–PCR; **P < 0.01 as compared with controls.

To verify whether γ-H_2_AX and 53BP1 were activated at telomeres, double immunofluorescence experiments were performed in U87 cells. Confocal microscopy revealed that most of the γ-H_2_AX foci and 53BP1 foci induced by BRACO-19 colocalized with TRF1 protein (Figure 3a–3c), forming the so-called telomere dysfunction-induced foci (TIFs) [27–30]. Quantitative analysis indicated that BRACO-19 significantly increased the percentage of cells with more than four γ-H2AX/TRF1 or 53BP1/TRF1 colocalizations (the percentage of TIFs-positive cells reached about 65% upon treatment; *P*< 0.01), with a mean of ca. 7 TIFs per nucleus (Figure 3b–3c). Notably, an important fraction of the damage response was not colocalized at telomeres. Analysis of human genome composition has identified that many G-quadruplex-forming sequences are located outside telomeres [31, 32]. Therefore, it is possible that BRACO-19 can also interact with other G-quadruplex targets. These results were confirmed by quantitative reverse-transcription PCR (qRT–PCR)-based chromatin immunoprecipitation (ChIP), as described previously [27, 28]. The ChIP assay showed that γ-H_2_AX and 53BP1 associated to telomeres in BRACO-19-treated cells (Figure 3d). These results demonstrated that BRACO-19 triggered DNA damage response specifically occurred at telomeric regions. These telomere-based DNA damage responses have also been reported for some G-quadruplex interactive agents [28, 33].

### Evidence of telomere uncapping induced by BRACO-19

**Figure 4:**
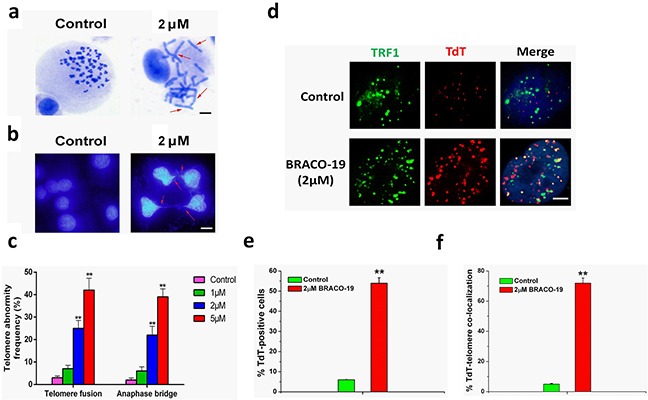
Telomere uncapping induced by BRACO-19 **a.** Telomere fusion induced by BRACO-19. Metaphase spreads were stained with Giemsa. Scale bar equals 10 μm. **b.** Representative images of anaphase bridges in U87 cells treated with BRACO-19 (2μM) for 72 hours were shown. Cells were stained with DAPI and images were recorded. Red arrow indicated bridge formation. Scale bar equals 10 μm. **c.** The frequency of telomere instability was calculated as the ratio between cells exhibiting anaphase bridges and the total number of anaphase cells (at least 50 anaphase cells were examined). Telomeric fusion frequency was calculated as total number of telomeric fusions/total number of metaphases. **P < 0.001. **d.** BRACO-19 induced accessible telomere ends. TRF1 (green) were used to detect telomeres, whereas TdT-cy3 (red) was used as a marker of uncapped telomeres in U87 cells treated with BRACO-19. Merged signals were shown in the right. Scale bar equals 2 μm. **e.** Quantification of the percentage of TdT-cy3-positive cells in BRACO-19 -treated cells. **f.** Quantification of the percentage of co-localization of telomeric signals with TdT-cy3 signals in BRACO-treated cells. In panels e and f, a minimum of 100 nuclei was scored, and error bars represented s.d. **P < 0.001.

A current model proposes that telomere forms ‘a cap’ at the end of chromosomes [1–3, 13]. It has been hypothesized that induction of quadruplex formation at the telomere may result in alterations of telomere capping, evidenced by the formation of anaphase bridges and fused telomere [28, 34]. Next we explored whether G-quadruplex stabilization induced by BRACO-19 could interfere with telomere integrity and induce formation of anaphase bridges. Telomere status was analyzed in U87 cells by staining of nuclei with DAPI, performed on 72h of treatment, and revealed that cells treated with BRACO-19 displayed typical images of anaphase bridges, which indicated telomere uncapping (Figure 4a–4b). Furthermore, metaphase spreads in the treated groups were also prepared and stained with Giemsa. As shown in Figure 4a–4c, remarked telomere fusion was observed in treated cells (*P*<0.001), indicating the chromosome abnormality. However, in normal primary astrocytes, treatment with BRACO-19 did not induce these multiple cytogenetic aberrations (Supplementary Figure S4). Moreover, to directly label the unprotected telomere, a terminal deoxytransferase (TdT) assay that added cy3-conjugated deoxyuridine to naked telomere ends was applied [27, 28]. The TdT-cy3 assay did not detect specific nuclear staining in untreated cells. However, in BRACO-19-treated cells, ~65% of TdT signals colocalized with telomeres, indicative of robust telomere uncapping (Figure 4d–4f). Taken together, these results demonstrated that BRACO-19 can induce telomere uncapping and expose chromosomal termini to the DNA-damage pathway.

### BRACO-19 induce T-loop disassembly characterized by the release of telomere-binding proteins from telomere

**Figure 5:**
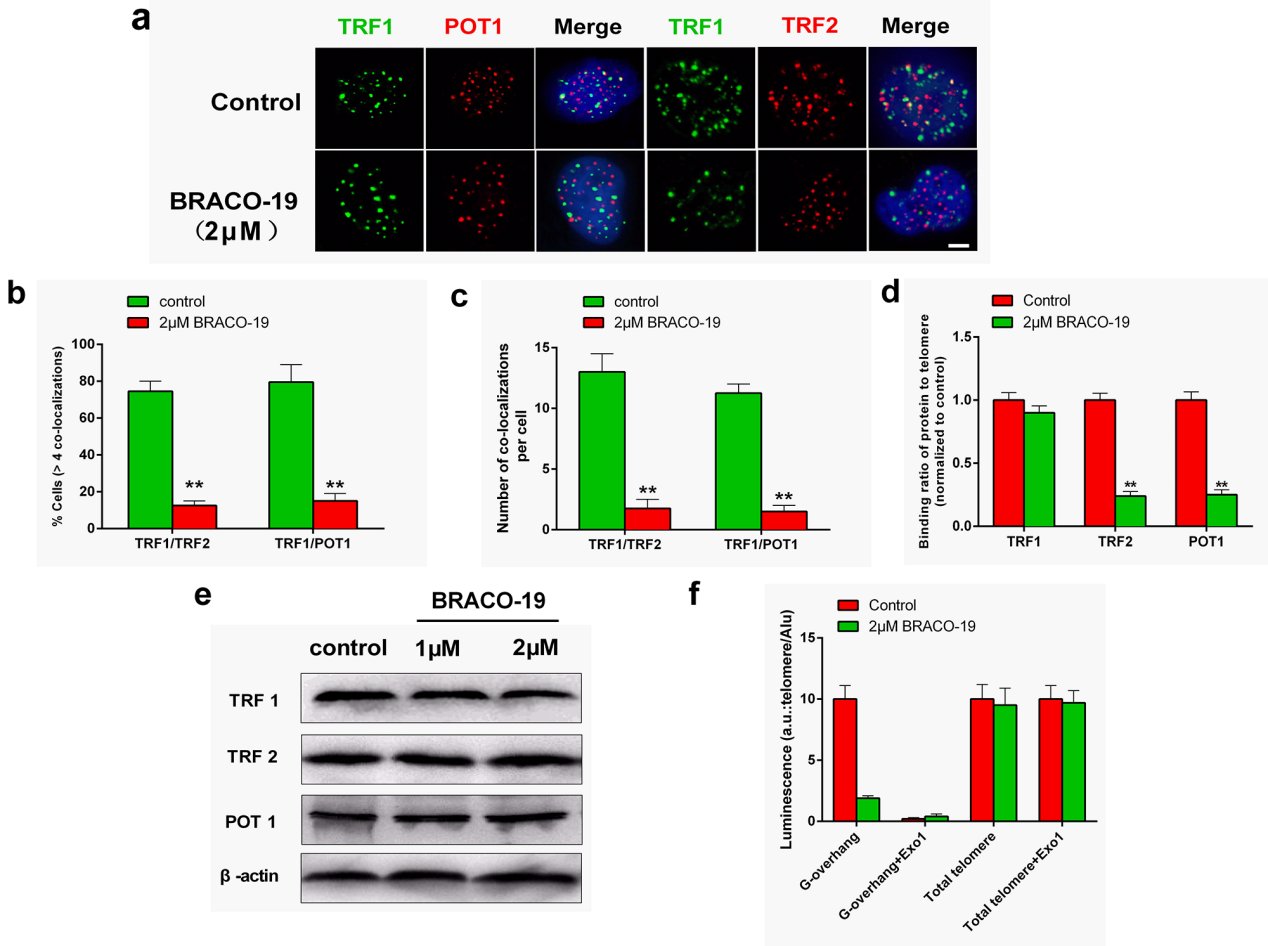
BRACO-19 specifically delocalizes TRF2 and POT1 from telomeres and induces telomeric 3′-overhang degradation **a.** U87 cells treated with BRACO-19 (2 μM) for 72 hours were double stained with the indicated antibodies. Representative confocal images showing merged TRF1 (green) with TRF2 and POT1 (red) staining in untreated and treated cells. Scale bar equals 5 μm. **b.** Percentages of cells with more than four co-localizations per nucleus of TRF1/TRF2 and TRF1/POT1. Error bars indicated s.d. **P < 0.005. **c.** Average number of co-localizations per nucleus in U87 cells treated with BRACO-19 (2 μM). Error bars indicated ± s.d. **P < 0.005, two-tailed student's *t*-test. **d.** Binding of TRF1, TRF2 or POT1 was examined by ChIP assay and detected by qRT–PCR amplification of the telomeric region in U87 cells treated with BRACO-19 (2 μM). Data represented triplicate ChIP experiments, each with technical triplicates of qRT–PCR; **P < 0.01 as compared with controls. **e.** Expression of TRF1, TRF2 and POT1 in U87 cells treated with BRACO-19 (2 μM). β-actin was used as loading control. **f.** Hybridization protection assay (HPA) was performed on genomic DNA isolated from U87 cells treated with BRACO-19 (2 μM) to assess the length of G-overhang and total telomere length. *ExoI* nuclease digestion was used to assess integrity of the 3′-overhang. Luminescence intensity in arbitrary units (AU) was normalized against *Alu* probe. Error bars indicated ± s.d., **P < 0.01, two-tailed student's *t*-test.

The telomere uncapping was usually associated with the dissociation of telomere-binding protein from telomere [9, 35, 36]. We next investigated the effect of BRACO-19 on the localization of TRF2 and POT1, two telomeric proteins that can induce telomere dysfunction and induce DNA damage signaling when their levels are reduced at telomeres [1–3, 29, 35]. Confocal microscopy showed that BRACO-19 specifically delocalized TRF2 and POT1 from TRF1 foci in U87 cells after 72 hours of treatment (Figure 5a). Quantitative analysis indicated that the percentage of nuclei with more than four TRF2/TRF1 or POT1/TRF1 co-localizations was markedly reduced in cells exposed to BRACO-19 (Figure 5b–5c). To confirm the results of these immunofluorescence analyses, we performed quantitative real time-polymerase chain reaction (qRT-PCR)-based ChIP assay as described above using the same antibodies used in the immunofluorescence experiments. As expected, BRACO-19 significantly reduced the binding of TRF2 and POT1 to the telomere, without affecting the association of TRF1 to the telomere, in agreement with the immunofluorescence results (Figure 5d). We also provided evidences that the removal of TRF2 and POT1 from telomere was not associated with the change of expression of these proteins (Figure 5e). Furthermore, we investigated the effect of BRACO-19 on telomeric G-overhang length and the total telomere length by using Hybridization Protection Assay (HPA) [27, 28, 34]. As shown in Figure 5f, BRACO-19 significantly reduced the telomeric G-overhang length after 72 hours of treatment (*P* < 0.01), whereas the total telomere length did not change. Meanwhile, we demonstrated that BRACO-19 did not induce POT1 and TRF2 delocalization and telomeric 3′-overhang degradation in normal primary astrocytes (Supplementary Figure S5). These results demonstrated that BRACO-19 can selectively induce T-loop collapse and reduce the telomeric G-overhang length in glioma cells, which indicate G-quadruplex formation [28, 34, 36].

**Figure 6:**
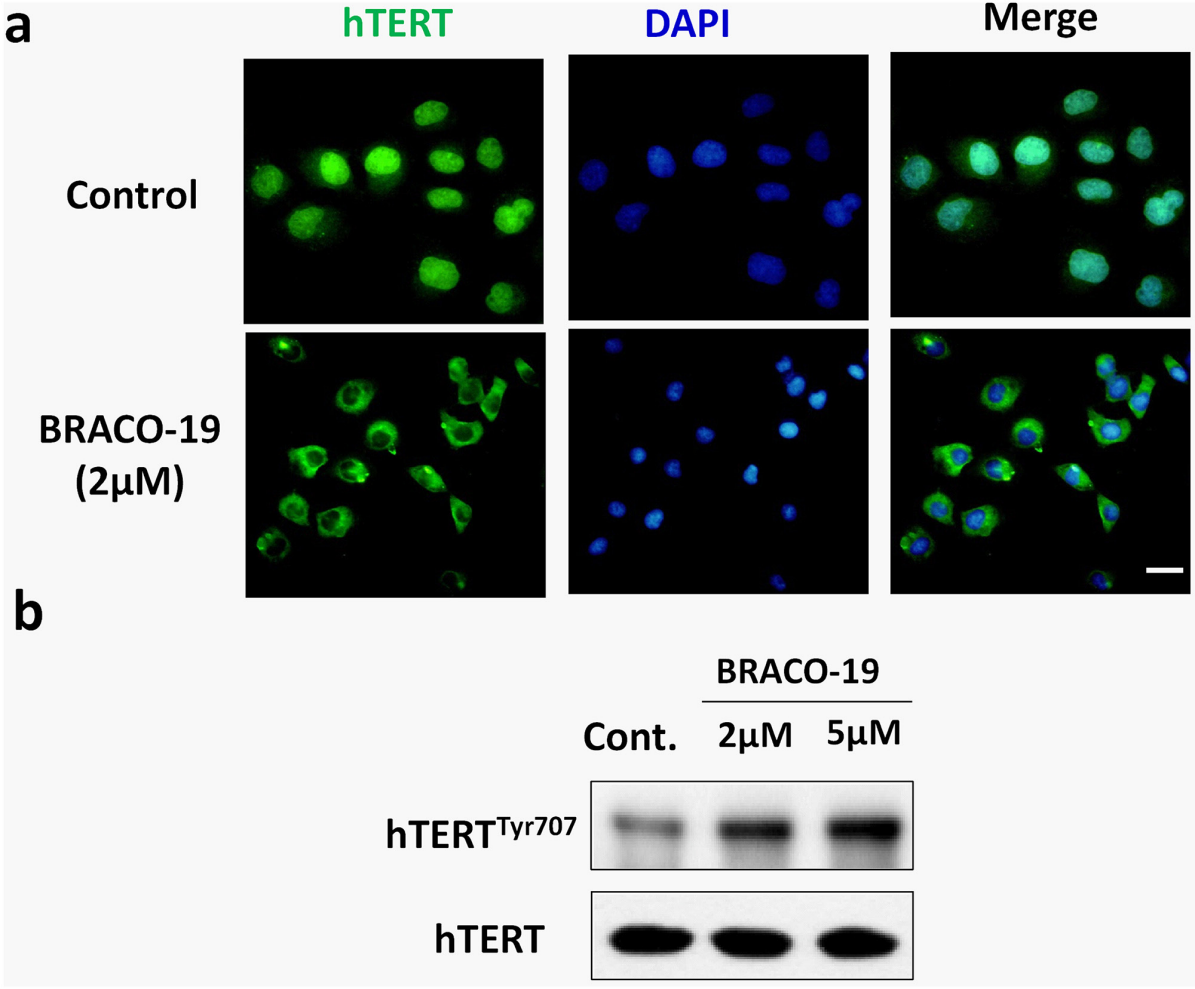
BRACO-19 treatment leads to a decrease of hTERT expression in the nucleus and translocation to cytoplasm **a.** U87 cells treated with BRACO-19 were stained with the hTERT antibodies for hTERT (green) and DAPI for nucleus (blue). Representative confocal images were shown. Scale bar equals 20 μm. **b.** Western blot analysis of hTERT phosphorylation in cells exposure to BRACO-19. The levels of hTERT were used as loading control.

Next, we explored the effect of BRACO-19 on the localization of telomerase. Immunofluorescence analyses revealed that after 72h treatment, telomerase (hTERT) translocated from nuclear to cytoplasm in treated-U87 cells (Figure 6a). It has been established that hTERT shuttling between subcellular compartments involved in telomerase activity regulation [37, 38]. Although the molecular mechanism regulating nuclear localization of hTERT is unclear, the Tyr^707^ phosphorylation is reported to regulate the subcellular location of hTERT [39]. As shown in Figure 6b, we found that the Tyr^707^ of hTERT was phosphorylated on exposure to BRACO-19, which may charge for the translocation of hTERT under this situation.

### Short-term apoptosis and senescence evoked by BRACO-19-induced telomere dysfunction

**Figure 7:**
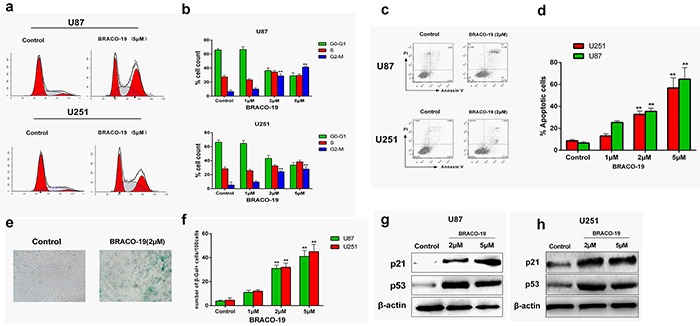
Cell cycle arrest, apoptosis and senescence evoked by BRACO-19-induced telomere dysfunction **a, b.** Cell cycle arrest induced by BRACO-19 in U87 and U251 cells. 72 hours after treatment with BRACO-19, cells were collected and stained with propidium iodide (PI); DNA content was determined by flow cytometry. **P < 0.01. **c, d.** Apoptotic cell death induced by BRACO-19 in U87 and U251 cells. 72 hours after treatment, cells were collected and stained with PI and Annexin V–FITC, Annexin V-positive/PI-negative cells were measured by flow cytometry. **P < 0.01. **e.** Representative images of SA β-gal positive cells in U87 cells that treated with BRACO-19 (2 μM) and control groups. Scale bar equals 50 μm. **f.** Expression of senescence-associated β-galactosidase (SA-β-gal) in U87 and U251 cells after treatment with BRACO-19 for 72 hours. The senescent cells were counted under an inverted microscope in five random fields. **P < 0.001. **g, h.** Upregulation of p53 and p21 proteins induced by BRACO-19. Immunoblotting for β-actin was performed to verify equivalent protein loading.

Furthermore, we explored whether telomere dysfunction induced by BRACO-19 resulted in cell cycle arrest, apoptosis or senescence [40–42]. We first analyzed the percentage of cells in different phases of the cell cycle. As shown in Figure 7a–7b, after 72 hours treatment, BRACO-19 induced significant accumulation of cells in the G_2_/M phase and concomitant decrease in the G_0_-G_1_ phase (*P* < 0.01).

Besides, we also observed BRACO-19-induced apoptosis and senescence. Annexin V assay was performed in U87 and U251 cells to assess apoptosis after treatment with BRACO-19 for 72 hours. As shown in Figure 7c–7d, apoptosis occurred after exposure to BRACO-19. The induction of apoptosis resulted from the inability of cells to pass the G_2_/M checkpoints. Moreover, the apoptosis was also accompanied by the occurrence of a senescence phenotype: large cell size, vacuolated cytoplasm and β-galactosidase activity. As shown in Figure 7e–7f, marked increase in the percentage of senescent cells was observed in 2 μM BRACO-19-treated cells (*P* < 0.001). However, control groups did not show these effects. The induction of apoptosis and accelerated senescence has been described as one of the characteristics of G-quadruplex-interacting ligands in cancer cells [28, 33–34, 43].

To address the molecular mechanism associated with growth arrest and accelerated senescence induced by BRACO-19, immunoblottings were performed to investigate changes in the expression of p53 and p21 proteins, which have been considered as key regulators of cell cycle and cellular senescence [28, 44, 45]. As indicated in Figure 7g–7h, after 72 hours exposure to BRACO-19, significant upregulation of p21 and p53 was observed in U87 and U251 cells, indicating that p21 and p53 were involved in the growth inhibition. The involvement of p21 and p53 in telomere-directed senescence has been sufficiently validated [28, 29, 45]. Based on these results, the G-quadruplex binding ligand BRACO-19 can not only inhibit telomerase, but also trigger a series of telomere-related cellular events and possesses selectivity against cancer cells.

### Overexpression of POT1 increases G-overhang and protects cells from BRACO-19 treatment

**Figure 8:**
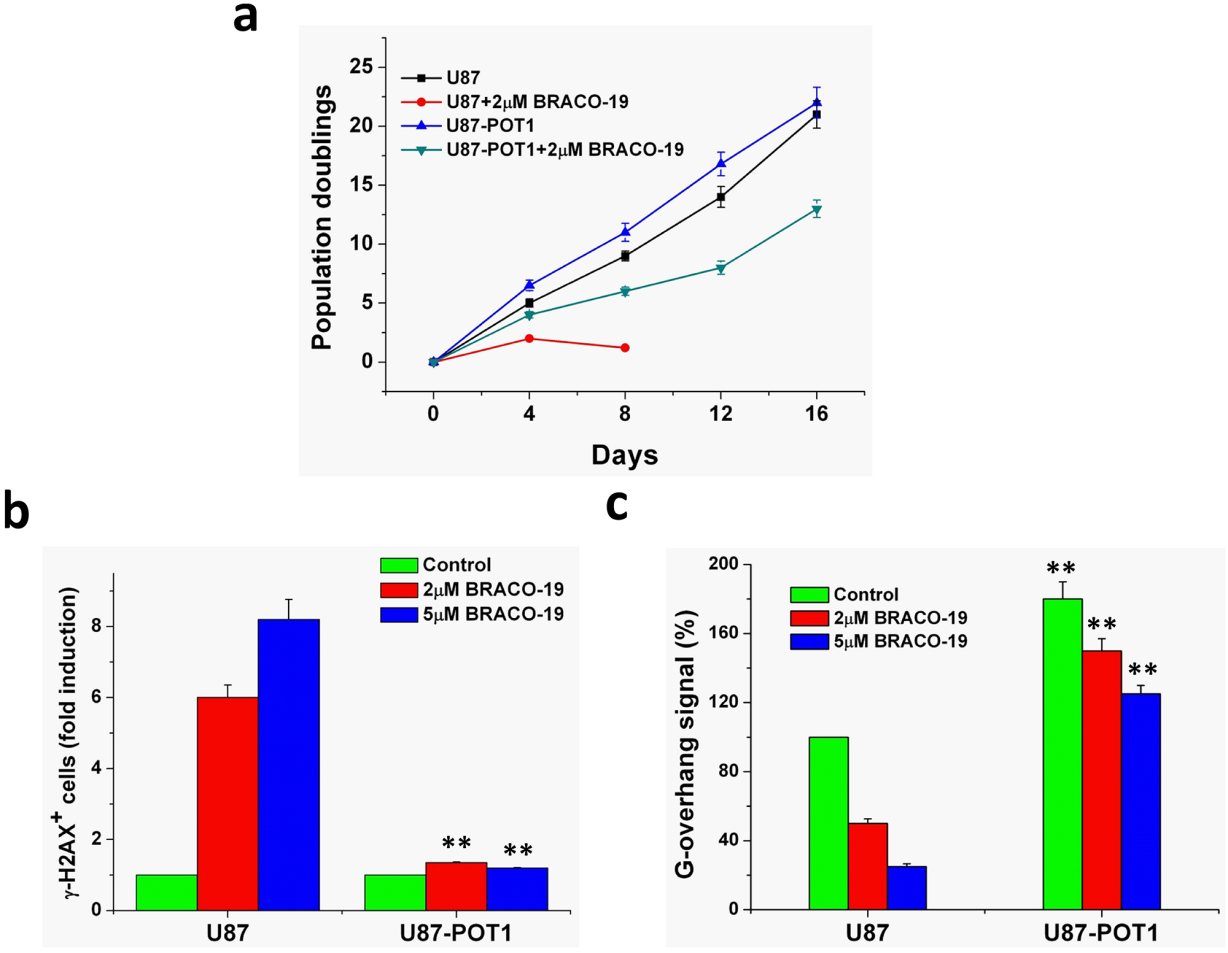
Overexpression of POT1 increases G-overhang and protects cells from BRACO-19 effects **a.** Proliferation curves of U87 and U87-POT1 cells treated with BRACO-19 (2μM). At the indicated times, cells were counted and the **Population doublings (PDs)** were determined. **b.** POT1 overexpression antagonizes BRACO-19 induced damage response. U87 and U87-POT1 cells were treated with BRACO-19 (2μM, 5μM) for 72h and processed for IF. Histogram represents the increase of γ-H_2_AX-positive cells compared to the untreated ones. Error bars represent s.d. **c.** G-overhang signal of U87 cells transfected with POT1 in the absence or treated with BRACO-19 (2 and 5μM) for 72 h. Hybridization protection assay (HPA) was performed on genomic DNA isolated from U87 and U87-POT1 cells to assess the length of G-overhang. Luminescence intensity in arbitrary units (AU) was normalized against *Alu* probe. Error bars indicated s.d.

POT1 protein is essential for telomere capping and allows us to regulate potential G-quadruplex structures formed at the telomeric G-overhang *in vitro* [46–48]. Overexpression of POT1 may protect or modulate the telomere dysfunction induced by G-quadruplex ligands. We therefore examined whether overexpression of POT1 could modulate the cellular effects of BRACO-19. Treatment of U87 cells with BRACO-19 induced a cell growth arrest after four population doublings, followed by cell death at day 5 (Figure 8a). Interestingly, U87-POT1 cells presented a noticeable resistance to the effect of BRACO-19 because the growth arrest was not observed after 15 days (Figure 8a). As a control, doxorubicin treatment of the cell lines did not indicate significant resistance in U87-POT1 cells, as compared with U87 (Supplementary Figure S6). These results indicated that reinforcement of telomere capping functions by POT1 counteracted the effects of BRACO-19 on telomeres and tumor cells.

To determine whether this protection corresponded to a difference in the effect of BRACO-19 at telomeres, we then analyzed DNA damage response and the G-overhang degradation in these two cell lines, U87 and U87-POT1. As shown in Figure 8b, γ-H_2_AX-positive cells were much lower in U87-POT1 cells compared with U87 cells after treatment with BRACO-19, suggesting that POT1 overexpression antagonizes BRACO-19 induced damage response. Moreover, we noticed that the G-overhang signal measured in U87-POT1 was much higher, as compared with U87 cells, indicating that the overexpression of POT1 had a positive regulatory effect on the G-overhang length (Figure 8c). Treatment of U87-POT1 and U87 cells with BRACO-19 for 3 days induced a dose-dependent reduction of the G-overhang signal. However, the decrease of the G-overhang signal in U87-POT1 cells was lower than that in the U87cells, and the remaining G-overhang signal was therefore higher than that in treated U87 cells. These results indicated that the POT1 expression protecting G-overhang from degradation played an important role in the resistance mechanism to BRACO-19 for U87-POT1cells. These results also indicated that BRACO-19 could target the telomere architecture and damage the G-overhang structure through stabilization of G-quadruplex [28, 34, 36, 49].

## DISCUSSION

Small molecules that target G-quadruplexes in telomeric DNA disrupt telomere maintenance in cancer cells and hence become attractive potential anticancer agents [17, 18]. Genetic-based validation studies have provided a compelling argument which suggests that the telomere maintenance pathway is a well validated target at the preclinical level [8, 9].

G4 ligands were initially evaluated as telomerase inhibitors, but their antiproliferative effect may occur quickly, before any telomere shortening takes place [20, 28, 50]. This short-term effect cannot be explained by telomerase inhibition, which would lead to a gradual shortening of telomeres after a certain number of cell divisions. The observations that G4 ligands delocalize telomeric proteins from telomeres, activate DNA damage response at telomeres, and induce chromosomal end-to-end fusions strongly suggest that their direct target is the telomere and not the telomerase enzyme [17, 18, 20].

The frequent detection of both chromosome end fusions in metaphasic nuclei and anaphase bridges confirmed that BRACO-19 act on telomeres and induce telomeric instability as in the case of deprotected telomeres [28, 34]. Dynek and Smith have demonstrated that telomeres of sister chromatids normally associate and that their resolution is required for progression through mitosis [51]. G-quadruplex ligands could thus also interfere with these steps by prevention of the correct conformation of telomeres and/or by inducing the formation of intermolecular G-quadruplex structures possibly favored by sister chromatid association.

Telomeric quadruplex formation induced by BRACO-19 has resulted in the removal of telomere-binding proteins TRF2 and POT1 from telomere, which has been identified to have important roles in telomere maintenance and end-protection [29, 35, 52]. The reason for that BRACO-19 do not affect another telomeric dsDNA-binding protein, TRF1 binding to telomere may be due to its different binding sites from TRF2 on telomere and different roles. TRF1 has a propensity for binding long tracts of dsDNA and has been postulated to modulate the length of telomere via its interaction with other telomere associated proteins [35, 53]. In contrast, TRF2 can directly bind not only the telomeric dsDNA but also the ds/ssDNA junction near the 3′-overhang, and promote the overhang to invade the upstream duplex region to form the T-loop structure [13, 54]. Moreover, TRF1 has been reported to have an approximately four times higher binding affinity to telomeric DNA than TRF2 [55]. Therefore, when the telomeric DNA forms quadruplex structure, TRF2 would be more sensitive and easily to dissociate from telomere than TRF1.

By contrast, the rapid removal of POT1 induced by BRACO-19 is likely to result at least in part from the inability of this protein to bind G4 structures [48], which, indeed, are stabilized by the drug. One might also speculate that BRACO-19 alters other telomeric components involved in the recruitment of POT1 at telomeres [46, 56]. They results are in agreement with previous findings showing that Telomestatin, another G4 ligand, delocalizes POT1from telomere [36].

Cells overexpressing POT1 become resistant to BRACO-19 suggests that telomere is the main target of BRACO-19, even if we cannot exclude that BRACO-19-induced damage could extend beyond the telomeric regions. In particular, it is tempting to speculate that G4 DNA is perceived as damage because it prevents t-loop formation and/or blocks the progression of the replication fork through telomeric DNA [33, 34, 57]. POT1 overexpression might counteract the deleterious effects of the stabilization of G4 DNA by BRACO-19 through several means, including the formation of t-loop that would mask the 3′-overhang against BRACO-19 binding [13, 35], resulting the elongation of this overhang; the recruitment and stimulation of RecQ helicases, which are expected to resolve G4 DNA or other DNA structures induced by the stabilization of G4 during telomere replication or recombination [58, 59].

Previous studies have demonstrated that quadruplex formation at telomere and concomitant telomere uncapping can activate some nucleases/exonucleases to process the ends, which result in rapid reduction of telomeric G-overhang signals [60, 61]; removal of telomere-binding proteins (TRF2 and POT1) from telomere can also cause loss of telomeric G-overhang [34, 36, 52]; Telomeric G-overhang degradation was found to be associated with the onset of replicative senescence or apoptosis in normal cultured cells [15, 40, 62], but also with telomere capping alterations [52]. A recent work also indicated that telomestatin induced G-overhang degradation (38–52% in human A549 cells treated for 8–12 days) in association with the onset of replicative senescence [36]. Signals initiated by telomere dysfunction are similar to those initiated due to double-strand DNA breaks, and they are typically mediated by the DNA damage response mediated ATM/ATR-p53–p21 pathway [33, 57]. Tumor cell apoptosis could be the linking factor between telomere instability, DNA damage response, and decreased proliferation [28, 40].

To determine the therapeutic window of BRACO-19, we need to address the question of whether or not the normal brain can tolerate doses of BRACO-19 required to kill tumor cells. We displayed that BRACO-19 did not induce the growth inhibition of normal human primary astrocytes, as well as the DNA damage response, telomere abnormality and T-loop disassembly. This was in line with previous studies with other G4 ligands showing that they did not affect the proliferation and viability of normal cells like fibroblasts [34, 63].

The selectivity of G-quadruplex ligands for cancer cells remains an intriguing issue in the telomere field. It cannot be excluded that protein composition at the telomere may differ, quantitatively and/or qualitatively, in normal versus tumor cells and that normal cells may be provided with a higher degree of telomere stability, thus becoming less sensitive to telomere-interacting agents [8, 9, 35]. In this scenario, the less sensitive C6 cells to BRACO-19 treatment may exhibit higher levels of POT1 protein at telomeres than other glioma cells [35, 46, 48]. Moreover, the selective effects could also result from a checkpoint failure that would allow the accumulation of deleterious DNA damage. The very early appearance of telomere damage and the subsequent detrimental effects on cell viability suggest that cells have to be chronically exposed to the drug to accumulate enough lethal damage [63]. Whatever the precise reason, this differential response is intriguing and may open new avenues of investigation.

These data reinforce the notion that these agents can act as inhibitors of telomere-related process and therefore the rationale for the development of this class of inhibitors as antitumoral agents. To our knowledge, this is the first report of BRACO-19-induced viability loss in brain tumor cells. Alone, or in combination with other treatments, such as radiotherapy, they may provide a new basis for the treatment of glioblastoma multiforme, as well as other types of cancers.

## MATERIALS AND METHODS

### Cell lines

U87 (human glioblastoma), U251 (human glioblastoma), SHG-44 (human glioma) and C6 (rat glioma) cell lines were obtained from American Type Culture Collection (ATCC).

### Telomere repeat amplification protocol (TRAP)

Telomerase activity was assessed in crude cellular extracts using TRAP assay with a Telomerase PCR ELISA kit (Roche) as described previously [26–28]. Briefly, telomerase was prepared from extracts of exponentially growing cells by lysing for 30 min on ice in a CHAPS-based buffer [0.5% w/w CHAPS, 10mM Tris-HCl, pH 7.5, 1 mM MgCl_2_, 1Mm EGTA, 5mM 2-mercaptoethanol,10% (v/v) glycerol]. The lysate was then centrifuged at 12,000g for 30 min at 4°C, and the supernatant was collected and stored frozen in aliquots at −80°C for up to 3 months. Total cellular protein was then determined, we assayed 500ng of protein extract in a 50ul reaction mixture containing 10 μl of 5×TRAP reaction mix and 2U of *Taq* DNA polymerase (TaKaRa). The reaction mixture was incubated for 45 min at 30°C for telomerase extension, and was then subjected to PCR amplification for 30 cycles of 94°C for 30 s, and 55°C for 30 s on a AMPLITRON^®^ Thermolyne (Alpha Multiservices, Inc). Amplified products were visualized on a 12% nondenaturing polyacrylamide gel, after electrophoresis and staining with 0.2% AgNO_3_. Images were photographed using a UVP gel documentation system (Ultraviolet Products, Upland, CA, USA). Each reaction product was amplified in the presence of a 36-bp internal standard. Telomerase activity was assessed by determining the ratio of the entire telomerase ladder to that of the internal control, using Lab works 4.5 image analysis software.

### ChIP assays

ChIP assays were performed was performed as previously reported [27, 28]. Briefly, cells were fixed in 0.8% paraformaldehyde in 1×PBS, washed extensively in 1×PBS, lysed in ice-cold Lysis buffer (1% SDS, 10 mM EDTA, 50 mM Tris–HCl at pH 8.0 and protease inhibitors; sonicated chromatin products of ~100–300bp) and diluted 10× in dilution buffer (20 mM Tris–HCl at pH 8.0, 150 mM NaCl, 2 mM EDTA, protease inhibitors and 1 mg ml ^−1^ BSA). ChIP was performed with the relevant antibody and captured with Protein A/G-Sepharose. DNA–protein complex was washed with 1× wash buffer I (20 mM Tris-HCl at pH 8.0, 150 mM NaCl, 0.1% SDS, 1% Triton X-100 and 2 mM EDTA), 2× wash buffer II (20 mM Tris–HCl at pH 8.0, 250 mM NaCl, 0.1% SDS, 1% Triton X-100 and 2 mM EDTA) and eluted in 1% SDS and 100 mM NaHCO_3_. Eluate fraction was de-cross-linked by high-salt treatment (200 mM NaCl) at 60°C followed by proteinase K treatment at 50°C. DNA extracted was subjected to PCR (see below).

### Standard and telomere PCR analysis

Telomeric sequences in immunoprecipitates were evidenced by PCR amplification according to a method described previously [27, 28]. The final telomere primer concentrations were 270 nM (tel1) and 900 nM (tel2), and PCR amplification was subjected to 35 cycles of 95°C for 15s, 54°C for 2 min. The primer sequences were as following: tel1 5′-GGT TTT TGA GGG TGA GGG TGA GGG TGA GGG TGA GGG T-3′and tel2 5′-TCC CGA CTA TCC CTA TCC CTA TCC CTA TCC CTA TCC CTA-3′. Quantitative PCR was done using the SYBR green Jumpstart Taq ReadyMix (TaKaRa) on a Roche LightCycler 480.

### Cytogenetic analysis

To determine the presence of anaphase bridges, cells were seeded on glass coverslips in complete culture medium and treated with BRACO-19 for one week, then stained with DAPI (Sigma) and mounted. Images of anaphases were recorded with an Olympus BX-51 fluorescence microscope (Tokyo, Japan) coupled with a CCD camera controlled by DP 70 software. The frequency of anaphase bridges was calculated as the ratio between cells exhibiting anaphase bridges and the total number of anaphase cells. At least 50 anaphase cells were examined in each experiment. Chromosome aberrations were evaluated as previously reported [34, 27, 28]. To obtain chromosome preparations, cells in the log phase of growth were incubated with 0.1μg/ml colchicine for 2h and trypsinized, then incubated with hypotonic 0.075 M KCl for 10 min, fixed with methanol/acetic acid (3:1, v/v), dropped onto frosted microscope slides, and air-dried overnight. Chromosomal aberrations were blindly evaluated by two independent observers in Giemsa- and DAPI-stained metaphases from two grown cultures for each treatment.

### Telomere TdT assay

Labelling unprotected telomeres with cy3-conjugated deoxy-Uridine (Amersham) was performed essentially as described [27, 28], except that the TdT incubation time was 20 min at 37°C to minimize background. Co-localization of the TdT signal with telomeres was performed by fixing TdT-labelled cells with 2% paraformaldehyde, washing three times in PBS, followed by incubation with anti-TRF1 antibody, and visualized with fluorescein-conjugated secondary antibody. Images were taken using an Olympus Fluoview FV1000 confocal microscope.

### G-tail telomere HPA

For Hybridization Protection Assay (HPA) [34, 27, 28], 1 μg non-denatured genomic DNA and 0.5 μg heat-denatured genomic DNA was used per assay for the detection of telomere 3′ overhangs and total telomere DNA, respectively. Briefly, 100μL of 3 × 10^6^ rlu (relative light units) of acridiniumester (AE)-labeled telomere HPA probe (5′-CCC TAA CCC TAA CC*C TAA CCC TAA CCC TA-3′; *AE position) in hybridization buffer was added to the DNA solution, mixed well by vortex, and incubated at 60°C for 20 min. Specific activity of AE-labeled probe was 8 × 10^7^ rlu/pmol probe DNA. Hydrolysis of the AE of unhybridized probes was carried out by adding 300 mL of the hydrolysis buffer to each reaction tube, mixing well by vortex, and incubating at 60° C for 10min. The AE of hybridized probe was not hydrolyzed under these conditions. The tubes were cooled on ice for over 1min, and chemiluminescence was measured for 2 s per tube with a luminometer (BPCL-2-TGC, Ultra Weak Luminescence Analyzer). For normalization of genomic DNA amount in each assay, we used *Alu* mouse repetitive HPA probe (5′-TGT AAT CCC A*GC ACT TTG GGA GGC-3′; *AE position). To check specificity of G-tail detection, non-denatured genomic DNA was treated with Exonuclease I (30 U/μg DNA) at 37° C for overnight, and heat inactivated at 80° C for 20 min, before G-tails were assayed. Probes for AE-labeling of telomeres and detection of Alu sequences were supplied by Bioneer Inc. (Korea).

### Statistical analysis

The data were expressed as means ± s.d. Statistical analysis was performed by Student's t-test (one- or two-tailed). The criterion for statistical significance was taken as *P < 0.05 or **P < 0.01.

Other related experimental methods, such as cell culture, cytotoxicity assays, immunofluorescence, immunoblotting, cell cycle, apoptosis, senescence, immunuprecipitation, plasmid construction, cell proliferation assay, and so on are available in Supplementary Methods.

## SUPPLEMENTARY METHODS FIGURES


