# Supplementary material for: Telomere targeting with a novel G-quadruplex-interactive ligand BRACO-19 induces T-loop disassembly and telomerase displacement in human glioblastoma cells
Source: Oncotarget. 2016 Feb 18;7(12):14925–39. doi: 10.18632/oncotarget.7483 (PMC4924762; doi:10.18632/oncotarget.7483)
Supplement: Supplementary file 1 [file oncotarget-07-14925-s001.pdf]

SUPPLEMENTARY METHODS

Cell culture and drug preparation

Cells were cultured in Dulbecco's modified Eagle's medium (DMEM, Gibco) supplemented with 10% v/v fetal bovine serum (FBS, Sigma), 1% w/v penicillin (10^4 U/ml)/streptomycin (10 mg/ml) (Sigma). Human primary astrocytes were isolated as described previously and cultured as above [64]. BRACO-19 were dissolved in pure water at a concentration of 2 mM to produce stock solutions, which were stored at -80°C. These solutions were diluted with culture medium immediately before use.

In vitro growth inhibition and cytotoxicity assays

Cells growth inhibition and cytotoxicity was detected by the conversion of MTT to formazan. Briefly, U87, SHG-44,C6 and U251 cells were seeded in 96-well plates at a concentration of 5000 cells/well and grown overnight at 37°C/5%CO_2, The BRACO-19 was added in 0.2 mL of medium to obtain final drug concentrations between 0.05 and 25μM. At 72h after treatment , the cells were incubated with 20μl MTT (5 mg/ml) for 4 h at 37°C. Then removing the supernatant and the formazan was solubilized in 150μl of DMSO. The plates were read at 490 nm with a Bio-Rad model-680 microplate reader.

Immunoblotting analysis

Immunoblotting was performed as described previously [26–28]. Cells after treatment were washed with PBS, and lysed in 100μM of lysis buffer (10 mM Tris-HCl, pH 7.4, 5 mM $MgCl_2$, 1 mM EDTA, 25 mM NaF, fresh 100 mM Na_3VO_4 and 1 mM dithiothreitol). Cell lysates were centrifuged for 20 min at 12000g. Concentrations of protein in the supernatant were determined by Bicinchoninic acid assay. Equal amounts of protein (40μg) were resolved on 12% SDS-PAGE, and transferred electrophoretically to PVDF membrane. The membranes were blocked with nonfat dry milk (5%) in PBST (10 mM Tris-HCl, pH 7.4, 150 mM NaCl, 0.05% Tween-20), and then incubated with primary antibodies overnight at 4°C, washed three times with PBST, and then incubated with secondary antibody (HRP-conjugated) for 2h at room temperature. After washing the secondary antibody, the bound antibody complex was detected using an ECL chemiluminescence reagent (Thermo). The primary antibodies used in this experiment were: mAb anti-TRF1 (Novus), mAb anti-TRF2 (Novus), pAb anti-POT1 (Sigma), mAb anti-γ-H2AX (Genscript), pAb anti-H2AX (Genscript), mAb anti-hTERT (Rockland), anti-$hTERT^{Tyr707}$ (Thermo), anti-p53 and anti-p21 (Santa Cruz), β-actin (Sigma).

Immunofluorescence

Immunofluorescence was performed as previously reported [27, 28]. Cells were fixed in 2% formaldehyde and permeabilized in 0.25% Triton X100 in PBS for 5 min at room temperature. For immunolabeling, cells were incubated with primary antibody and then washed in PBS and incubated with the fluorophore-conjugated secondary antibodies. The following primary antibodies were used: mAb anti-TRF1 (Novus), mAb anti-TRF2 (Novus), pAb anti-POT1 (Sigma), mAb anti-γ-H_2AX (Genscript), pAb anti-H_2AX (Genscript), mAb anti-53BP1 (Novus), mAb anti-hTERT (Rockland). The following secondary antibodies were used: Rhodamine or DyLight ™488 conjugated goat anti-rabbit, fluorescein or DyLight™594 conjugated goat anti-mouse (Jackson Laboratory). Fluorescence signals were captured by using Olympus Fluoview FV1000 confocal microscope and analyzed by FV10-ASW 1.6 Viewer program (Olympus, Japan).

Cell cycle analysis

Briefly, after 72h treatment with BRACO-19, cells (0.5-1×10^6) were washed with PBS, fixed in 70% ethanol and kept at -20°C for at least 24 h. Then cells were washed in PBS and resuspended in 50 μg/ml propidium iodide and 100 μg/ml RNase in PBS. The cell suspension was incubated for 30 min at room temperature in dark and cell cycle distribution was determined by flow cytometry (FACSCalibur, Becton-Dickinson), with CellQuest software.

Apoptosis analysis

Surface exposure of phosphatidylserine in apoptotic cells was measured using an AnnexinV/FITC and PI apoptosis detection kit (BD). The cells were collected and resuspended in the binding buffer provided in the kit, then mixed with PI and FITC conjugated Annexin V. After incubation for 15 min, the cells were assessed via flow cytometric analysis.

SA-β-Gal assay

The senescent cells were verified by staining for SA-β-Gal as described previously [26–28]. Cells treated with BRACO-19 were wvashed twice in PBS, fixed in 2% formaldehyde/0.2% glutaraldehyde for 5 min at room temperature, washed again in PBS, and incubated for 16 h with β-Gal stain solution containing 1mg/ml 5-bromo–4–chloro-3-indolyl-β-D- galactoside, 40 mM citric acid / sodium phosphate (pH 6), 5 mM potassium ferrocynide, 5 mM ferricyanide, 150 nM NaCl and 2 mM $MgCl_2$. Cells were viewed with a OLYMPUS BX-51 light microscope and photographed.

Construction and transfection of POT1 over-expression plasmids

pLPC-NMYC-POT1 were taken as the template plasmids, which was obtained from Addgene (plasmid 16069, de Lange Titia, Rockfeller University). The POT1 gene was amplified by PCR with primers containing EcoRI and BamHI sites and cloned into the corresponding cloning sites in the pcDNA3.1/mycmammalian expression vector. The plasmids were transfected with Lipofectamine 2000 (Invitrogen) following the manufacturer's instruction. Forty-eight hours after transfection, positive transfected cells were selected with 600 μg ml^{-1} G418. The Myc tag was detected by western blotting.

Cell proliferation assay

Cells were seeded in growth medium into T80 tissue culture flasks at 1.25×10^5 cells per flask and exposed to various concentrations of BRACO-19 every 2 days. The cells in control and BRACO-19-treated flasks were trypsinized and counted using a hematocytometer, and the flasks were reseeded with 1.25×10^5 cells per flask. For cells stably expressing POT1, same concentrations of cells were seeded, and every 2 days, the cells were trypsinized and counted, and flasks were reseeded with the same number of cells. Results were expressed as the cumulated population doubling as a function of the time of culture as described previously [26–28].

REFERENCE

64. Pennarun G, Granotier C, Gauthier LR, Gomez D, Hoffschir F, Mandine E, Riou JF, Mergny JL, Mailliet P, and Boussin FD (2005) Apoptosis related to telomere instability and cell cycle alterations in human glioma cells treated by new highly selective G-quadruplex ligands. Oncogene 24:2917–2928.

SUPPLEMENTARY FIGURES

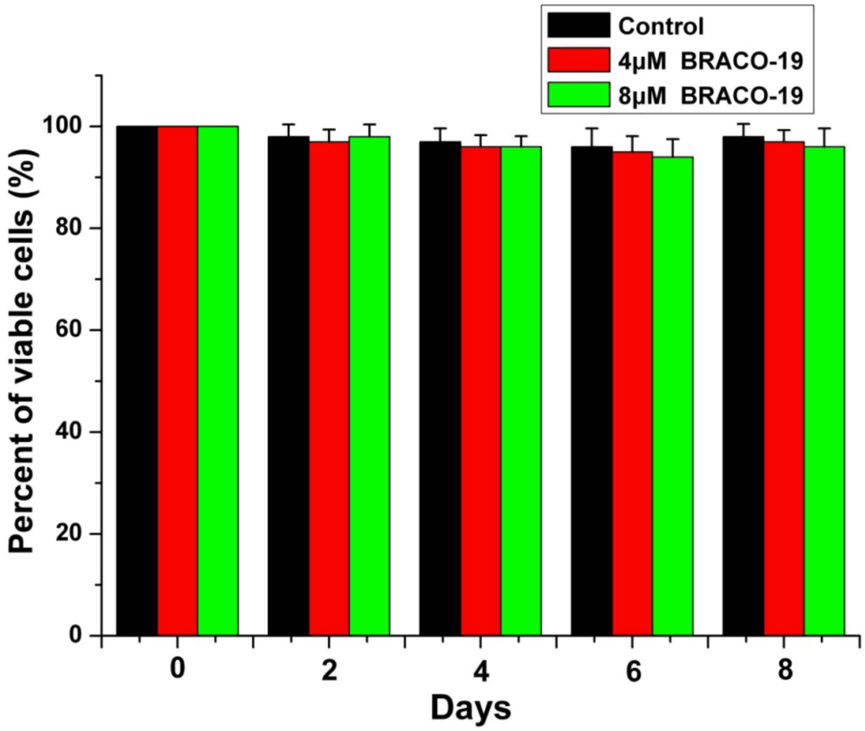

Supplementary Figure S1: Survival rates of BRACO-19-treated human normal primary astrocytes. Percentages of viable cells after BRACO-19 (4 µM and 8 µM) treatments at indicated days are shown. At the indicated times cells were counted by using the hematocytometer and the viability was determined by trypan blue dye exclusion. The mean of three independent experiments with comparable results is shown.

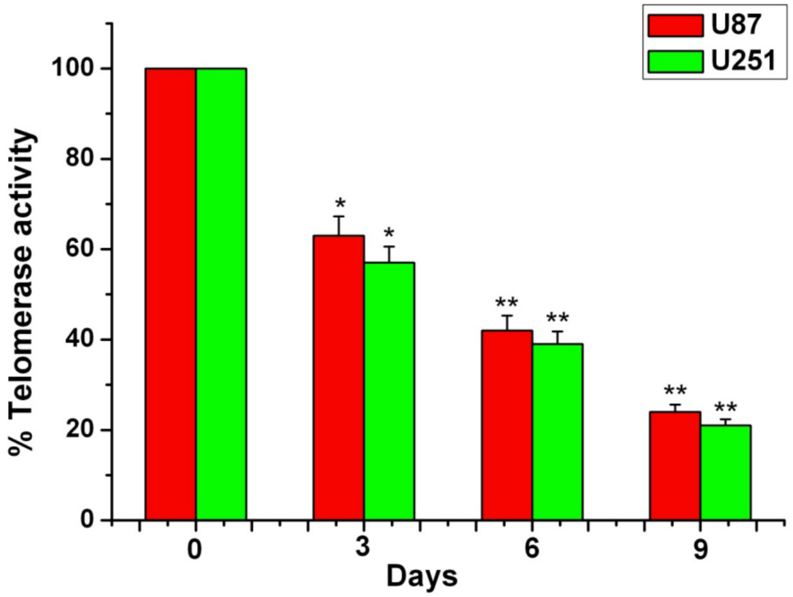

Supplementary Figure S2: Time course of telomerase inhibition induced by BRACO-19 in U87 and U251 cells. Time course of telomerase inhibition induced by BRACO-19 at 2μM in U87 and U251 cells. Cells were treated with BRACO-19 for 3, 6, 9, and 12 days, respectively. CHAPS extract was prepared at indicated time-points and equivalent amounts of protein (500 ng) were subjected to a standard TRAP assay. Enzyme activity is represented as the percentage of untreated sample at time zero. The mean of three independent experiments with comparable results is shown. Error bars indicate ± SD, n=3. *$P<0.05$, **$P<0.01$, one-tailed *t*-test.

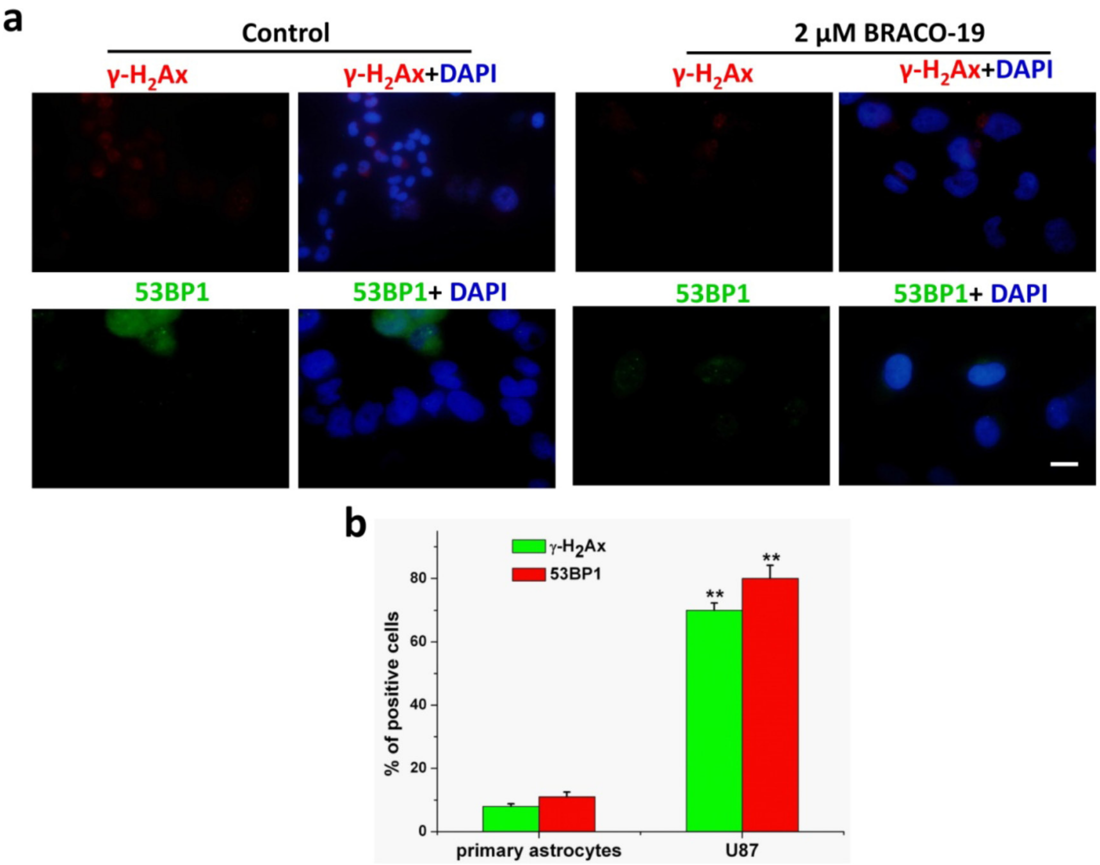

Supplementary Figure S3: BRACO-19 does not induce damage in human normal primary astrocytes. Human normal primary astrocytes were treated with BRACO-19 for72h, fixed and processed for IF. **a.** Representative immunofluorescence images of γ-H2AX and 53BP1 foci in primary astrocytes treated with BRACO-19. Scale bar equals 15 μm. **b.** The histogram represents the percentage of γ-H2AX and 53BP1-positive cells in primary astrocytes and U87 cells. The mean of three independent experiments with comparable results is shown. Error bars indicate ± SD, ** $P<0.01$, one-tailed *t*-test.

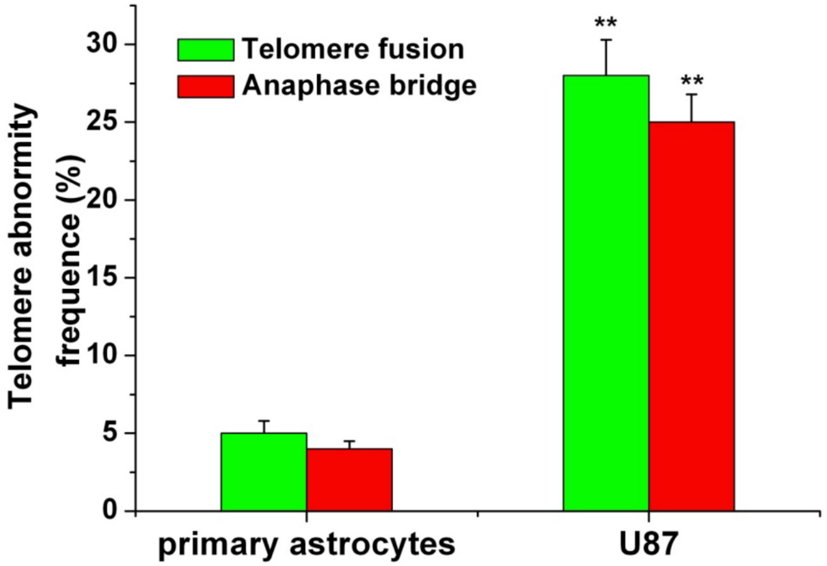

Supplementary Figure S4: BRACO-19 does not induce telomere instability in human normal primary astrocytes. Primary astrocytes and U87 cells were treated with BRACO-19 (2 µM) for 72 hours, Cells were stained with DAPI and metaphase spreads were prepared. Telomere fusion and anaphase bridges images were recorded. The frequency of telomere instability was calculated as the ratio between cells exhibiting anaphase bridges and the total number of anaphase cells (at least 50 anaphase cells were examined). Telomeric fusion frequency was calculated as total number of telomeric fusions/total number of metaphases. The data represented the means of three independent experiments with s.d. **$P < 0.001$.

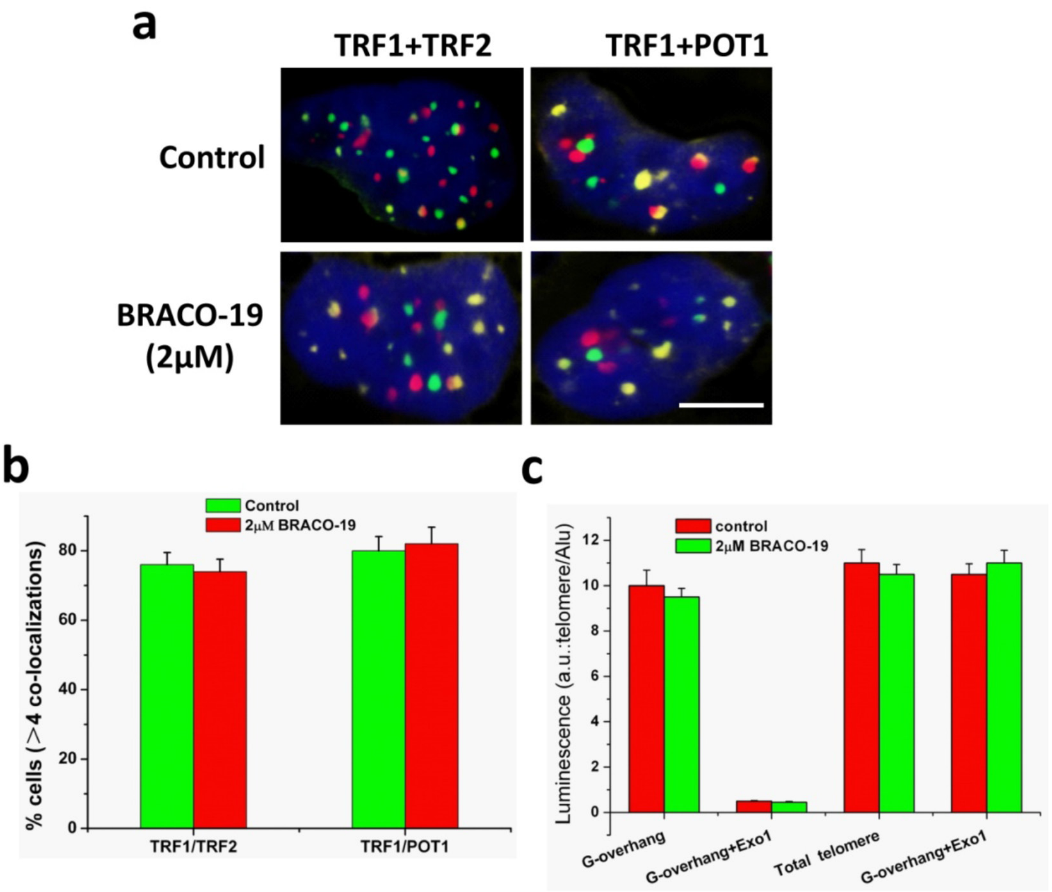

Supplementary Figure S5: BRACO-19 does not induce POT1 and TRF2 delocalization and telomeric 3′-overhang degradation in normal primary astrocytes. a. Primary astrocytes treated with BRACO-19 (2 µM) for 72 hours were double stained with the indicated antibodies. Representative confocal images showing merged TRF1 (green) with TRF2 and POT1 (red) staining in untreated and treated cells. Scale bar equals 10 µm. **b.** Percentages of cells with more than four co-localizations per nucleus of TRF1/TRF2 and TRF1/POT1. Error bars indicated s.d. $**P < 0.005$. two-tailed student's *t*-test. **c.** Hybridization protection assay (HPA) was performed on genomic DNA isolated from primary astrocytes treated with BRACO-19 (2 µM) to assess the length of G-overhang and total telomere length. *ExoI* nuclease digestion was used to assess integrity of the 3′-overhang. Luminescence intensity in arbitrary units (AU) was normalized against *Alu* probe. Error bars indicated ± s.d., $**P < 0.01$, two-tailed student's *t*-test.

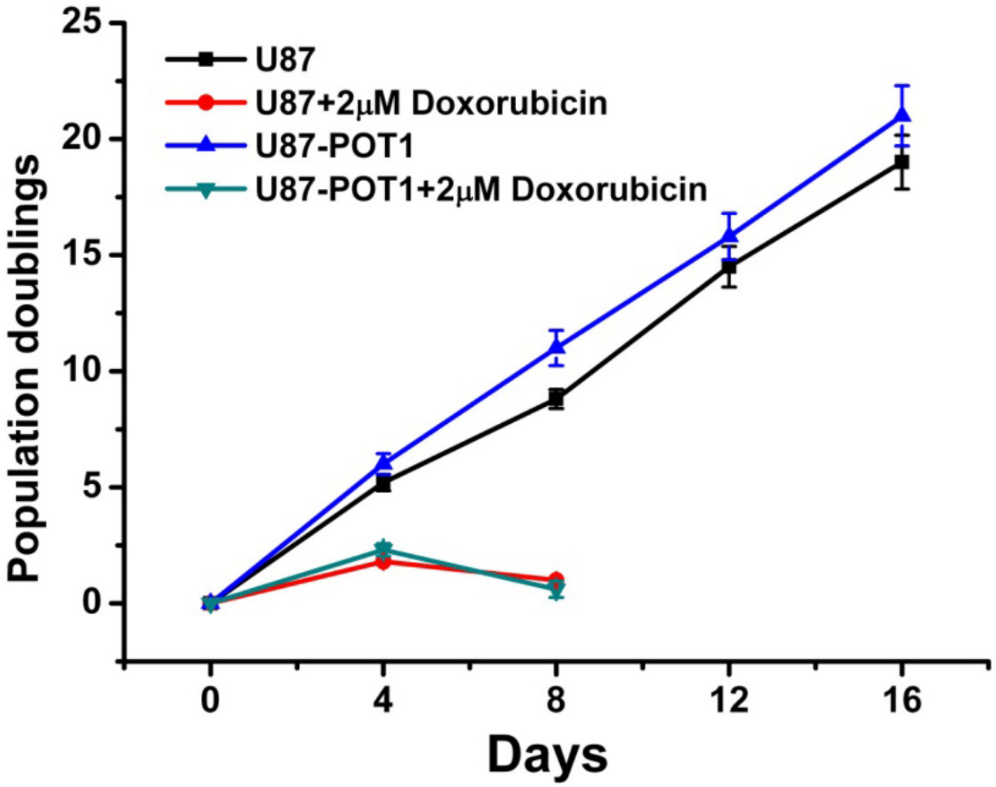

Supplementary Figure S6: Overexpression of POT1 did not increase resistance against doxorubicin in U87 cells. Proliferation curves of U87 and U87-POT1 cells treated with doxorubicin (2μM). At the indicated times, cells were counted and the (Population doublings, PDs) were determined.
